# MDMA for the Treatment of Negative Symptoms in Schizophrenia

**DOI:** 10.3390/jcm11123255

**Published:** 2022-06-07

**Authors:** Mitchell D. Arnovitz, Andrew J. Spitzberg, Ashkhan J. Davani, Nehal P. Vadhan, Julie Holland, John M. Kane, Timothy I. Michaels

**Affiliations:** 1 Department of Psychiatry, The Zucker Hillside Hospital, Northwell Health, Queens, NY 11004, USA; marnovitz@northwell.edu (M.D.A.); aspitzberg@northwell.edu (A.J.S.); adavani@northwell.edu (A.J.D.); nvadhan@northwell.edu (N.P.V.); jkane2@northwell.edu (J.M.K.); 2 Department of Psychiatry, The Donald and Barbara Zucker School of Medicine at Hofstra/Northwell, Hempstead, NY 11549, USA; 3 Institute of Behavioral Science, Feinstein Institutes for Medical Research, Manhasset, NY 11030, USA; 4 Private Practice, New York City, NY 10016, USA; jholland@inch.com

**Keywords:** negative symptoms, schizophrenia, psychosis, MDMA, psychedelic-assisted therapy

## Abstract

The profound economic burden of schizophrenia is due, in part, to the negative symptoms of the disease, which can severely limit daily functioning. There is much debate in the field regarding their measurement and classification and there are no FDA-approved treatments for negative symptoms despite an abundance of research. 3,4-Methylenedioxy methamphetamine (MDMA) is a schedule I substance that has emerged as a novel therapeutic given its ability to enhance social interactions, generate empathy, and induce a state of metaplasticity in the brain. This review provides a rationale for the use of MDMA in the treatment of negative symptoms by reviewing the literature on negative symptoms, their treatment, MDMA, and MDMA-assisted therapy. It reviews recent evidence that supports the safe and potentially effective use of MDMA to treat negative symptoms and concludes with considerations regarding safety and possible mechanisms of action.

## 1. Introduction

Schizophrenia is a complex psychiatric syndrome characterized by psychosis, or loss of touch with reality. Individuals with schizophrenia can suffer from positive symptoms, including hallucinations, delusions, and disorganized speech or behavior, as well as negative symptoms, including deficits in affect, speech, motivation, and sociality [1]. Schizophrenia can be difficult to treat, and the mean proportion of patients who achieve recovery is estimated to be as low as 13.5% [2]. Those living with schizophrenia have an increased risk of premature mortality, with an estimated near 29 years of life lost on average and suicide rates approaching 5% [3,4,5]. They are at a higher risk for developing various chronic medical conditions, with suboptimal recognition and treatment. Examples include coronary artery disease and metabolic abnormalities such as diabetes, cancer, respiratory illnesses, and stroke [2,5]. The health, social, and economic burden of schizophrenia vastly overshadows its prevalence of 2–6 per 1000 [6]. The total economic burden of schizophrenia is estimated to be over USD 60 billion per year in the United States [7].

Clinically significant negative symptoms are estimated to be present in approximately 60% of patients with schizophrenia in community and research samples [8,9]. For many it is the first symptom to manifest in the prodromal phase, well before the first psychotic episode. Negative symptoms continue to persist despite antipsychotic treatment [9] and correlate with increased disease severity and poorer functioning [8]. Negative symptom burden is associated with poor response to treatment and related functional outcomes [10]. It is abundantly clear that negative symptoms confer a significant financial burden on society and increased utilization of primary care [11,12]. To date, there are no approved treatments for negative symptoms anywhere in the world. 

3,4-Methylenedioxy methamphetamine (MDMA) is a schedule I substance with a controversial history and resulting stigmatization. In recent years, it has emerged as a novel therapeutic given its ability to enhance social interactions, generate empathy, and induce a state of metaplasticity in the brain [13,14]. MDMA has been shown to be an effective treatment for PTSD and is currently in phase III clinical trials [15]. As such, it is being explored as a treatment for other indications. The present review will detail how MDMA may also be uniquely qualified to target negative symptoms in schizophrenia.

## 2. Negative Symptoms

The terms “positive” and “negative” symptoms can be traced back to the mid-1800s, when British neurologists distinguished between distortions and deficits of motor and cognitive functions [16]. Over time, this became increasingly applied to mental disorders, eventually being described by Kraepelin as irreversible and progressive deficits in individuals with dementia praecox [17]. Shortly after coining the term “schizophrenia,” Bleuler emphasized negative symptoms as the core process of the illness, speculating that symptoms were rooted in the weakening of mental functions [18]. In the early 1980s, the first negative symptom scale was developed, paving the way for its conceptualization as a distinct domain [19]. Over the next few decades, additional tools were developed, leading to various constructs. It became increasingly clear that negative symptoms research was limited by a lack of consensus [17]. As such, an NIMH-sponsored meeting was convened in 2005, leading to the construct that predominates to this day [20,21].

Blunted affect refers to the reduction in observed emotional expression that is independent of an individual’s subjective experience of emotion. It is widely accepted that individuals with schizophrenia show reduced emotional expressivity regardless of medication status, for both positive and negative emotions [22]. This has been supported by research examining alterations in responsible muscle groups [23]. Relatedly, analyses of verbal and nonverbal expression among those with schizophrenia have demonstrated consistent results, independent of medication and internal states [24,25].

Alogia refers to the decrease in quantity and spontaneity of speech as rated by the clinician. Alogia has often been associated with various aspects of cognition, particularly verbal fluency [21]. Deficits in verbal fluency are highly correlated with and specific to alogia, suggesting a possible shared mechanism [26]. Although several aspects of speech have been shown to be impaired in individuals with schizophrenia, the most significant seem to be in production, particularly pauses [27]. Given the significance of speech in role and social functioning, alogia remains an important consideration in schizophrenia research. 

Anhedonia refers to the diminished capacity to feel pleasure. It has long been considered central to both schizophrenia and depression. Given this overlap, efforts have been made to characterize different aspects of anhedonia that may contribute to each spectrum of illness. It was traditionally thought that depression reduces an individual’s ability to experience pleasure from ongoing activities (consummatory anhedonia), whereas schizophrenia limits an individual’s ability to anticipate future pleasure (anticipatory anhedonia). However, this characterization has been challenged in recent literature, as depressed patients are also known to experience anticipatory anhedonia [28]. Adding to the complexity of the construct are various confounders, including source of information, comorbid depression, motivation, and cognition [22,29,30,31]. As a result, recent studies have failed to demonstrate differences between individuals with schizophrenia and healthy controls, emphasizing the need to further narrow the construct and related measures [32]. 

Avolition refers to reduced initiation and persistence of goal-directed activity, as reported and observed. Substantial efforts have been made to understand it, as it is the domain most intricately linked to functional impairment [33]. Relatedly, such deficits have been shown to be a predictor of functional outcome in individuals with schizophrenia [34]. Despite their preserved ability to experience pleasure relative to healthy controls, there is a known deficit in behaviors to obtain pleasure [35,36]. As such, there has been greater emphasis on reward prediction mechanisms, particularly as they relate to learning and executive function [35,37,38].

Asociality refers to the reduction in social initiative resulting from decreased interest and motivation to form relationships. Research has shown that this deficit is remarkably similar to that seen in autism spectrum disorders, particularly in those with predominantly negative symptoms [39]. As a construct, asociality has been the most challenging to study, given the contribution of related factors, including positive symptoms, comorbid depression, cognition, functional impairment, and lack of opportunities [21]. Research has even implicated avoidance as an important mechanism despite not being part of the construct [40]. Although the true etiology of asociality has yet to be elucidated, associations have been made with social cognition, dysfunctional beliefs, and the oxytocin system [41,42,43]. 

### 2.1. Secondary Negative Symptoms 

**Figure 1:**
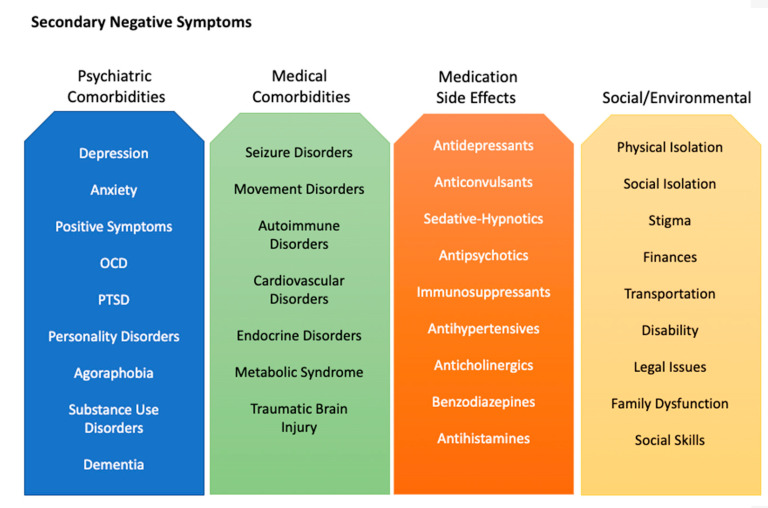
Actionable factors that cause or contribute to secondary negative symptoms.

Secondary negative symptoms refer to negative symptoms that are not intrinsic to schizophrenia. Secondary negative symptoms can be multifactorial and also respond to treatment [44]. They can arise from positive symptoms, depression, PTSD, dementia, substance use, or anxiety [44,45]. For instance, an individual with paranoid or persecutory delusions may be asocial and withdraw further from society due to fear, or an individual with concurrent PTSD may feel overwhelmed in crowds and similarly withdraw from social interactions. This is etiologically distinct from the negative symptom construct described above. Secondary negative symptoms can also arise from medical factors, such as insomnia, comorbid illnesses, and even medications (Figure 1). Patients with schizophrenia are commonly on antipsychotic medications, which are frequently associated with sedation, attenuation of motivation, extra-pyramidal motor disturbances, and other unpleasant side effects [44,45]. For these reasons, antipsychotic medications themselves may create a secondary cause of negative symptoms. The related changes in weight, libido, and appearance may also exacerbate pre-existing difficulties with relationships. Causes of secondary negative symptoms are difficult to disentangle from their primary negative counterparts in clinical trials, making measurement of treatment response even more complicated [44,45]. It is important for assessments to distinguish negative symptoms from external factors and those inherent to an individual’s schizophrenia. Thoughtful and appropriate data collection and measurement are thus of utmost importance in study design.

Although the measurement of negative symptoms has significantly progressed over the past four decades, it remains a complex challenge and one of the biggest limitations in the development of effective therapeutics. Psychometric assessments have been the traditional modality for measuring negative symptoms and have evolved alongside our understanding of negative symptoms. However, analyses of these scales have revealed that each has its own set of concerns [46]. As a result, efforts have shifted toward the development of digital tools to target specific objective constructs more precisely. For example, vocal analysis has emerged as a useful measure of schizophrenia severity and meta-analyses have demonstrated its utility in targeting negative symptoms [27,47]. Research has also suggested the utility of facial analysis in the measurement of negative symptoms and correlation with clinical ratings [48,49]. Tools have been developed to capture nonverbal behavior and have been shown to have excellent psychometric properties [50]. Given the impact of negative symptoms and cognition on recall in individuals with schizophrenia, ecological momentary assessment has emerged as a novel method of capturing valid data [51]. A final consideration of measurement is that of utilizing informants when available, as informant-based assessments have been shown to be among the most reliable predictors of functioning [52,53]. 

### 2.2. Cognition and Negative Symptoms 

The challenge of measuring negative symptoms may also be attributed, in part, to continued difficulties distinguishing the boundaries between negative symptoms, neurocognition, and social cognition. The relation of negative symptoms to cognitive deficits in schizophrenia has been a topic of substantial debate in the field for decades given that distinguishing the two has important implications for treatment targets and novel therapeutic development [54]. Cognitive deficits and negative symptoms are highly correlated with one another, and both are strongly associated with functional impairment [55,56]. Recent evidence examining the underlying neural mechanisms of both cognitive deficits and negative symptoms provides some evidence for shared pathophysiology, as both are associated with aberrant interhemispheric connections, and several genetic high-risk variants are strongly associated with negative symptom and cognitive deficit severity [57,58]. Both domains also share etiological risk factors, including neuroinflammation and oxidative stress [59,60]. Given this overlap, some have argued that measuring neurocognition and social cognition may be a more direct assessment of negative symptoms rather than relying on clinician-administered or patient self-report scales [61,62]. Although a comprehensive overview of the overlap of cognition and negative symptoms is outside the scope of the current review, we will briefly touch upon the role of cognition by highlighting two areas within neurocognition and social cognition that provide the strongest evidence of overlapping construct with negative symptoms. 

Patients with schizophrenia demonstrate reliable deficits across several domains of neurocognition, ranging from attention and processing speed to working memory and executive functioning difficulties [10,63,64]. These neurocognitive deficits are considered to be fundamental to the disease and have a disproportionate contribution to negative functional outcomes [64]. Perhaps the domain that most closely overlaps with the motivational deficits that characterize negative symptoms are impairments in effort-based decision-making. Patients with schizophrenia reliably demonstrate impairments in the willingness to exert both physical and cognitive effort [61]. Animal models have demonstrated that organisms choose to exert a minimal level of physical or cognitive effort to obtain a given outcome; however, most animals will increase effort when the reward is increased [65]. In contrast, patients with schizophrenia demonstrate effort-based impairments on these tasks [66,67]. Effort-based decision-making impairments are strongly associated with negative symptom measures, especially in patients who report negative beliefs about their own abilities and performance [40,68]. Tasks such as the Effort Expenditure for Rewards Task have been shown to have especially strong psychometric properties, including good test–retest reliability [62]. Therefore, measuring effort-based decision-making may serve as a strong correlate of motivational deficits in negative symptom clinical trials. 

Social cognition is comprised of various abilities that span lower levels of processing such as visual and auditory emotion recognition, to higher-order abilities such as theory-of-mind, empathy, and social connection [69]. Although some have argued that social cognition is more closely related to neurocognition than negative symptoms, such findings have often measured lower-level social cognitive processes such as facial and vocal emotion perception, and do not include assessments of naturalistic higher-order processes such as empathy or mentalization [70]. Deficits in empathic accuracy may be an especially relevant treatment target for considering the overlap of social cognition and negative symptoms given that empathy requires monitoring, perceiving, and interpreting the emotions of others and involves almost all of the brain regions involved in all other domains of social cognition [71]. Impairments in the ability to accurately monitor and interpret the social cues of others may help to explain the asociality component of negative symptoms. The Empathic Accuracy Task has been successfully implemented in previous schizophrenia clinical trials and may be especially beneficial for negative symptom assessment [72]. 

Clinical trials of negative symptoms will undoubtedly require some measurements of neurocognitive or social cognitive processes that overlap with the negative symptom construct. Not only do these measures help to address the aforementioned limitations of self-report and clinician-administered ratings of negative symptoms, but they also provide a direct objective measure of the components that contribute to anhedonia, asociality, and avolition. Therefore, it is strongly beneficial to incorporate relevant cognitive tasks into clinical trials for negative symptoms. 

### 2.3. Treatments for Negative Symptoms 

Treatments for negative symptoms to date include antipsychotics, antidepressants, stimulants, mood stabilizers, glycine and NMDA modulating agents, transcranial magnetic stimulation (TMS), oxytocin, and psychotherapy. Each of these has been investigated thoroughly but none have demonstrated efficacy, and each has various limitations. Antipsychotic medications are approved for the treatment of schizophrenia, following decades of research demonstrating their effectiveness for acute and chronic psychosis as well as relapse prevention [73,74]. However, these medications have largely been unsuccessful at producing clinically meaningful improvement in primary negative symptoms [75]. Although some studies have suggested the superiority of second-generation antipsychotics in the treatment of negative symptoms, there has been concern about funding bias and confounding depression [76]. More recent studies have shown that a newer antipsychotic, cariprazine, was superior to risperidone in treating negative symptoms. However, the measured effect size was small and both groups experienced a worsening of positive symptoms, demonstrating challenges for clinical implementation [77].

There has been substantial research on the augmentation of antipsychotic medications with antidepressant medications for the treatment of negative symptoms. Although meta-analyses have demonstrated statistical significance, their effect sizes have been relatively small and restricted to first-generation antipsychotics [78]. The effects also appear to vary significantly among the various agents studied. Multiple studies have attempted to utilize stimulants, agonists, or promoters of dopamine release to treat negative symptoms in patients with schizophrenia. Methylphenidate, amphetamine, and modafinil have all been studied in this context. The literature details the safety of these medications in this population, noting no worsening of positive symptoms in patients who were stable and effectively being treated on their antipsychotic medication regimen. Recent studies suggest that stimulant medication augmentation may improve negative symptoms, but the research is limited and at times inconsistent [79]. Anticonvulsant medications, including carbamazepine, valproic acid, lamotrigine, and topiramate, have all been investigated in their ability to treat negative symptoms as augmenting agents. Although one meta-analysis suggested a possible benefit of lamotrigine augmentation of clozapine, more recent investigations and meta-analyses have failed to find any benefit of these medications in treating negative symptoms [80,81].

In recent years, oxytocin has been demonstrated to play a central role in bonding and pro-social behavior, which has spurred interest in its use as a therapeutic in schizophrenia [82]. Numerous studies have associated lower plasma and CSF levels of oxytocin in patients with prominent negative symptoms [43,83]. Moreover, it has been demonstrated that low oxytocin release significantly correlates with negative symptoms in trust-related interactions [84]. However, meta-analyses of placebo-controlled RCTs investigating intranasal oxytocin have not shown any significant improvements [85,86]. 

The glycine receptor is heavily involved in NMDA receptor communication, which has been implicated in schizophrenia. Agents that interact with the glycine receptor include glycine, D-methylglycine, N-acetyl-cysteine (NAC), D-serine, and D-cycloserine. Multiple meta-analyses and randomized clinical trials have investigated these agents, as well as other novel agents that modulate glycine re-uptake, such as biopterin. Thus far the research has been inconclusive, with multiple contradictory results and no clear benefit from any of these agents [80,87,88,89]. Memantine, an NMDA receptor antagonist, has shown promise in treating negative symptoms [80]. Multiple small studies and case-reports have shown a positive effect of memantine augmentation on negative symptoms [90,91]. Although the effect sizes in these studies are small, there does appear to be consistency in the few published trials, warranting future study. 

Transcranial Magnetic Stimulation (TMS) is a neuromodulation technique in which magnetic pulses of varying strength and frequency are applied to targeted regions of the brain [92]. With the incorporation of functional and anatomical imaging data, treatment can be specific to altered neurocircuits in schizophrenia. However, the published data on TMS for schizophrenia are inconclusive as to its effect on negative symptoms [80]. As TMS treatment parameters and neuroimaging targets continue to be refined, further investigation is needed to elucidate its viability as a treatment option [92]. 

Therapy for negative symptoms can be broken up into three categories: skill-focused interventions, psychological interventions, and family interventions. Skill-focused interventions include social skills training, where participants learn techniques to respond to social cues and communication styles. It is one of the more widely studied interventions for negative symptoms and has been found to have a small superiority over other therapeutic interventions in meta-analyses [93]. Cognitive remediation, another skill-based therapy, has not been shown to be effective at treating negative symptoms. This is consistent with more recent literature, as cognitive deficits in schizophrenia are now believed to be functionally distinct from negative symptoms, as described above, likely warranting different interventions [94]. The most well-known psychological intervention is Cognitive Behavioral Therapy for Psychosis (CBTp), which was initially developed to treat positive symptoms [95]. This therapy functions similarly to other cognitive behavioral therapeutic styles, targeting distorted negative beliefs about social interactions. Meta-analyses have been able to show a significant effect of CBTp on negative symptom measures, but these data were secondary outcomes of studies not designed with negative symptoms as a primary outcome. More recent studies with study designs primarily focused on the measurement of negative symptoms have had mixed results [80,96]. Family interventions focus on assisting family members and providing support for them to act as a therapeutic entity for the patient. This support can come in many forms, and usually can include communication training, education, and crisis management. Many studies demonstrate that this added layer of familial support has a positive effect on a patient’s negative symptoms [94,97,98].

In conclusion, negative symptoms are a difficult entity to define, measure, and treat. Our current treatment modalities are limited, and there are currently no FDA-approved medications for negative symptoms. Novel pharmaceutical treatment modalities should thus be considered.

## 3. MDMA

MDMA is a psychoactive compound structurally similar to both amphetamine and mescaline that does not exist naturally and is created by laboratory synthesis [99]. It is considered an entactogen, a class of compounds that promote acceptance and compassion, modulate emotional responses, and augment interpersonal relationships and closeness [100,101]. MDMA has been illegal in the United States since 1985, when the DEA placed an emergency ban on the compound [102]. 

MDMA was incidentally discovered in Merck, Germany in 1912. It was synthesized as a byproduct in attempts to create a compound to stop abnormal bleeding [103]. In the decades that followed, MDMA was increasingly studied for its pharmacological properties, its structural similarities to epinephrine, and its behavioral effects [104]. Research had suggested that MDMA could allow a patient to engage openly in discussion about relationships and internal struggles, seemingly incapacitating prior defenses, prompting mental health practitioners to incorporate it into their practice [102]. Despite preliminary evidence of therapeutic potential, MDMA research was suppressed after being rescheduled by the DEA in 1986 given concerns about its increasing recreational use [100,102,105]. It has remained a schedule I substance ever since. 

### 3.1. Pharmacology

**Figure 2:**
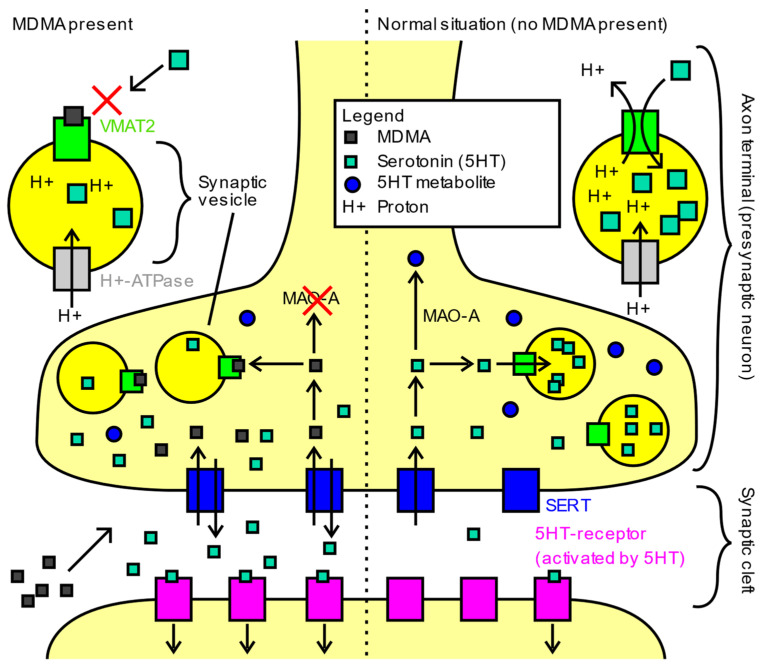
Main mechanisms of action of MDMA in humans. Image obtained from https://commons.wikimedia.org/wiki/File:Main_mechanisms_of_action_of_MDMA_in_humans.svg (accessed on 1 May 2022) and reproduced with permission under the Creative Commons CC0 1.0 Universal Public Domain Dedication.

MDMA acts on the monoamine system in multiple ways. It is a serotonin (5-HT), norepinephrine (NE), and dopamine (DA) reuptake inhibitor, and can even reverse reuptake, resulting in more release of neurotransmitter [106]. These effects are further compounded by vesicular monoamine transporter 2 (VMAT2) inhibition, which disrupts the packing of neurotransmitters into vesicles (Figure 2). This leads to increased cytosolic monoamines that can be shunted out of the cell by the reversed reuptake transporters noted above. MDMA has also been observed to inhibit monoamine oxidase A (MAO-A), resulting in decreased breakdown of monoamine neurotransmitters. MDMA can act as a receptor agonist at the 5-HT1A, 5-HT2A, 5-HT2B, and 5-HT2C serotonin receptors, as well as the alpha-1 and alpha-2a adrenergic receptors, D1 and D2 dopamine receptors, M1 and M2 muscarinic receptors, and H1 histamine receptors [106]. Through interactions with these receptors, MDMA has been shown to increase levels of cortisol, vasopressin, and oxytocin [107]. Through modulation of oxytocin release, MDMA has been found to evoke metaplasticity, a brain state in which new connections can be formed, leading to a reopening of the critical period of social reward learning, bolstering social and neuroplastic changes [14,108].

Serotonin has been implicated in playing a significant role in mental health [109]. Pharmacologic interventions commonly target serotonin for modulation in treating affective disorders, as seen in selective serotonin reuptake inhibitors (SSRIs). Newer antipsychotic medications, which also act as mood stabilizers, use a blockade of serotonin receptors to modulate dopamine release throughout the brain [110]. Psilocybin, a classical psychedelic shown to be effective in treating depression, acts as an agonist at serotonin receptors [111]. As such, it is speculated that serotonin may be involved in the therapeutic mood-altering effects of MDMA [112,113,114].

The importance of the 5-HT1A receptor on the effects of MDMA was studied by Kuypers et al. They used pindolol, which partially antagonizes the 5-HT1A receptor. In their study, MDMA and MDMA + pindolol both resulted in significant increases in oxytocin and cortisol release when compared to placebo, with no differences in social interactions and cognitive measures between the two treatment groups [115]. Although this study could not definitely prove that the pro-social effects of MDMA were not mediated through 5-HT1A, as the limited 5-HT1A occupancy of pindolol still allows for ~60% binding, they could not appreciate any diminishment of the pro-social effects of MDMA with a 5-HT1A blockade. This is supported by prior studies of similar design targeting 5-HT1A’s role in MDMA [116].

Given its role in the therapeutic action of various psychedelic compounds, the 5-HT2A receptor has been investigated as a possible mechanism for the pro-social effects of MDMA. Multiple studies have co-administered ketanserin, a 5-HT2A/C antagonist, with MDMA to elucidate the role of the 5-HT2A receptor [114,116]. 5-HT2A blockade appears to attenuate the acute physiologic and psychologic effects of MDMA, suggesting that it may be important in its therapeutic effects. However, unpublished animal model data from the lab of Gul Dölen indicate that a 5-HT2A blockade does not prevent a reopening of the critical period of social reward learning, a metaplastic state that is theorized to be a mechanism through which MDMA acts therapeutically in PTSD [117]. This suggests that although 5-HT2A is important for an individual’s acute experience, it may not mediate the underlying and long-term neuroplastic effects of MDMA. 

### 3.2. Drug Interactions

Given the abundance of serotonergic binding in MDMA, there exists a reasonable concern of co-administration with other serotonergic compounds. For example, using SSRIs in conjunction with other potent serotonergic agents increases the risk of serotonin syndrome, an overload of the serotonin system resulting in multiple physiologic abnormalities requiring immediate medical care [118]. Although serotonin syndrome has not been observed in the countless participants in the MDMA phase I, II, and III clinical trials for the treatment of PTSD, their serotonergic psychiatric medications were discontinued prior to treatment in these studies [15]. In addition, citalopram, an SSRI, was observed in studies to markedly reduce both physiological and psychological effects of MDMA after administration. Competitive binding of serotonin transporter (SERT) and competitive binding of endogenous serotonin are the likely mechanisms through which SSRIs disrupt the effects of MDMA [113]. Both for efficacy and safety, it is worth considering avoiding MDMA use in any patient currently on serotonergic medications [118]. 

Second-generation antipsychotics, by definition, utilize 5-HT2A antagonism to modulate dopamine blockade. In addition, these compounds also contribute to the blockade of numerous other serotonin receptors [110]. As noted above, although the data are not conclusive, many postulate that the pro-social effects of MDMA may be mediated through serotonin modulation. Because second-generation antipsychotics may block the serotonergic mechanisms through which MDMA acts, MDMA may be less efficacious for individuals taking second-generation antipsychotics.

Most first-generation antipsychotics (FGAs), unlike their second-generation counterparts, do not have any significant serotonergic blockade, and thus allow other serotonergic medications to modulate serotonin uninterrupted [119]. As such, one could speculate whether a first-generation antipsychotic would be less likely to inhibit the serotonergic and pro-social effects of MDMA. Although no studies have looked at MDMA therapy in individuals on an SGA, one study found that pretreatment with haloperidol, an FGA, did not alter the subjective experience of participants [120]. 

### 3.3. Safety Concerns

MDMA was criminalized in the 1980s due to concerns of dependance and neurotoxic effects [102]. Studies of MDMA administration in animal models demonstrated neuronal damage in the form of serotonergic axon degeneration, 5-HT depletion, and neuron terminal fragmentation. However, these studies administered MDMA in the form of repetitive high doses, between 10–20 mg/kg, mimicking binges of the substance at doses higher than the normal human recreational consumption of 1–3 mg/kg [121,122]. Throughout the years, researchers continued to cite concerns of neurotoxicity and cardiotoxicity, commonly pointing to the top percentile of Ecstasy users’ self-reported data, ignoring that “Ecstasy” refers to the street name of MDMA, which contains pure MDMA only ~3% of the time [123,124,125]. More recent studies of MDMA safety have been able to elucidate some of the true risks of MDMA use. In the various phase I–III clinical trials, MDMA has been well tolerated, with rarely any reported effects lasting beyond 24 h post-treatment [126]. 

An FDA-approved double-blind placebo-controlled phase I study in 1994 first set out to determine the safety of MDMA in healthy volunteers [127]. They determined that MDMA may lead to statistically significant, although transient and well-tolerated, increases in heart rate, blood pressure, and body temperature [127,128]. These physiologic findings were confirmed by subsequent trials that again demonstrated that the side effects of MDMA were generally well-tolerated [112,129,130,131]. As of May 2022, MDMA has been safely administered to over 1700 research subjects, in both phase I, II, and III studies [132]. In all phase I–III MDMA studies only one serious adverse reaction was reported, ventricular extrasystole exacerbation, in a participant who had an abnormal baseline EKG with noted ventricular extrasystole. The participant was thoroughly evaluated and returned to baseline within 24 h. No long-term sequalae were identified [126].

Given the prevalence of recreational Ecstasy use, cardiotoxic risks of MDMA have been described in the literature. These include atherosclerosis, ventricular dysfunction, and valvular heart disease. The metabolism of MDMA has been demonstrated to induce oxidative stress on ventricular myocytes in rats, correlating with case reports of contraction band necrosis in humans [133,134]. Valvular heart disease is thought to be mediated through MDMA’s activity at 5-HT2B receptors on cardiac interstitial cells. In a study looking at living human subjects, MDMA use was associated with valvular disease in a dose-dependent manner, implying that nonchronic use may confer lower risk [135]. This is consistent with the aforementioned data showing no such events occurring in phase I–III studies on MDMA-assisted therapy [126,132]. However, given that the cardiovascular system is most implicated in MDMA-associated fatalities, this theoretical consideration should be accounted for in future study designs [136].

The mechanism of MDMA-associated neurotoxicity, which has been observed in animal models, has not been fully elucidated [137]. One theory posits that serotonin depletion and SERT alterations, notably affecting the longer axonal projections of serotonergic neurons from the raphe nuclei, may be the route of neurotoxicity [138]. The data in humans have been equivocal, with many studies incorporating flawed methodology and confounding polysubstance use, and relying exclusively on retrospective self-reports of drug use outside of supervised lab administration [137]. To date, one meta-analysis of molecular imaging in drug users confirms that there may be neuroadaptive changes in SERT in Ecstasy users; however, they did not conduct meta-regression for correlations between drug use, lifetime dose, recency of use, or frequency of use [138]. In addition, several animal studies have found similar loss of SERT function and altered 5-HT terminals from chronic use of other serotonergic agents such as sertraline, paroxetine, and reserpine, yet they are not considered neurotoxins [122,139,140,141]. In fact, studies utilizing high-dose SSRIs observed neuroadaptive changes indistinguishable from those seen in MDMA [141]. Other similar human studies utilizing molecular imaging of SERT found that observed neuroadaptive changes are likely reversible after cessation of MDMA use [142].

Adding to the fear and stigma of MDMA, there have been multiple case reports and anecdotes of individuals adversely reacting to the substance with a psychotic break. Many of these reports are born out of illicitly acquired Ecstasy, with potential co-administration of other substances and no clarity of the purity or contamination of the Ecstasy in question [131,143,144,145]. To date, none of the MDMA therapy phase I, II, and III clinical trials have reported psychotic adverse events [146]. Although individuals with schizophrenia may be at higher risk for experiencing a psychotic symptom reaction, concurrent treatment with antipsychotics should provide adequate protection, as they do from other potential stressors [147]. 

Recently, a 2022 study demonstrated auditory–sensorimotor–thalamic hyperconnectivity in healthy volunteers following LSD, MDMA, and amphetamine administration [148]. This pattern of dysconnectivity has been compared to a similar pattern observed in individuals with schizophrenia [149]. In this study, MDMA induced salience–thalamic hypoconnectivity at the ventrolateral thalamic nuclei. In contrast, the salience–thalamic dysconnectivity observed in schizophrenia was at the mediodorsal nuclei [148]. This distinction is important to note, as MDMA did not recreate the neurocircuitry patterns of schizophrenia, as implied by the prior observations. Amphetamine induces a similar dysconnectivity pattern to MDMA and carries a similar dopaminergic profile [148]. Numerous studies have examined the use of psychostimulants (amphetamine, methylphenidate) in schizophrenia; significant exacerbation of psychotic symptoms in individuals actively treated with antipsychotic medications was not observed [150,151,152,153]. It thus stands to reason that MDMA may be safely administered to individuals with schizophrenia in active treatment with antipsychotics. Moreover, as described above, the use of an FGA pretreatment prior to MDMA administration in healthy volunteers did not attenuate the subjective experience compared to non-pretreated individuals and may help protect against unwanted adverse effects related to an increase in dopaminergic tone [120].

### 3.4. Current Applications of MDMA

The Multidisciplinary Association of Psychedelic Studies (MAPS) was created in 1986 in response to the criminalization of MDMA. This public corporation’s mission was to reveal the therapeutic potential of MDMA. In response to their foundational research that has since taken place, the FDA has designated MDMA-assisted therapy as a breakthrough treatment for PTSD. MDMA is currently undergoing its second phase III clinical trial for treatment of PTSD symptoms [132].

In the first phase III trial, 88% of participants with severe PTSD experienced a clinically significant reduction in PTSD diagnostic scores two months after their third session of MDMA-assisted therapy, compared to 60% of placebo participants. A total of 67% of participants in the MDMA group, compared to 32% in the placebo group, no longer met the diagnostic criteria for PTSD two months after their treatments [15,146].

Given the success of MDMA therapy for PTSD, MAPS expanded their focus to related disorders, including anxiety and eating disorders [132]. A recent study examined MDMA therapy as treatment for social anxiety in adults on the autism spectrum [104,154]. In this study, participants on the autism spectrum who underwent MDMA therapy had significant improvement in social anxiety symptoms that were maintained up to 6 months after study completion, as compared to the placebo group. Although this study had a small sample size of only 12 participants, the effect size was considerably large. The study also reported that two individuals from the MDMA therapy group spontaneously initiated social interactions in the form of romantic dating for the first time in their lives after completion of the treatment [154]. This study not only demonstrated a positive effect of MDMA in individuals on the autism spectrum, but also suggested its safety in this population. 

## 4. MDMA in Schizophrenia

The potential for MDMA in the treatment of negative symptoms in schizophrenia primarily revolves around its potential for targeting social function and reward processing. As described above, there is an abundance of evidence that MDMA increases sociality and can augment personal relationships and closeness [100,102,104,105,154]. MDMA has the potential to at the very least elicit an episode of closeness with a practitioner, thereby facilitating a strengthening of therapeutic alliance that may lead to improved care and outcomes [155]. Schizophrenia and autism have much in common, with similar neuroimaging and clinical findings [156]. In autism, reduced connectivity in subcortical regions, including the nucleus accumbens, has been observed and is associated with social deficits [157]. MDMA has benefited this population, and although negative symptoms were not the treatment target, the resulting changes in behavior were observed to be pro-social [104,154]. In schizophrenia, decreased volume of subcortical regions, notably the nucleus accumbens, has been observed to be evident as early as the first episode of psychosis [158].

Negative symptoms have been theorized to be in part related to decreased dopamine transmission in the mesocortical system and its connections to and from the nucleus accumbens and prefrontal cortex [44]. In animal studies, MDMA has been shown to induce metaplasticity in the nucleus accumbens, leading to a reopening of the critical period of social reward learning and the formation of new synaptic connections [14]. This occurs via oxytocin-mediated long-term depression, which has been observed to require coordinated serotonergic activity [159].

It stands to reason that Increased plasticity of the nucleus accumbens would result in an increased number of synaptic processes and a strengthening of the connections to and from this region. This could therefore enhance dopaminergic signaling to and from the nucleus accumbens, which has been theorized to be one method of ameliorating negative symptoms [44]. More specifically, the prefrontal network of reward processing, driven by dopaminergic reinforcement signals from the nucleus accumbens, requires sustained neural firing within a short temporal window [160]. Through its ability to increase monoamine release, MDMA may be able to strengthen the sustained neural firing required for intact reward processing [161]. This augmentation of neuronal firing could in theory be longstanding through the mechanisms of increased plasticity described above. This would thereby reduce the deficit in reward processing commonly seen in patients with schizophrenia.

Studies that only focus on oxytocin or serotonergic systems independently, as in the case of intranasal oxytocin and antidepressants, respectively, may miss the full potential of therapeutic benefit due to the lack of coordinated serotonin and oxytocin system activation in the nucleus accumbens. MDMA, with its inherent oxytocinergic and serotonergic effects, is poised for therapeutic benefit in negative symptoms.

To date, no lab studies or clinical trials have been conducted that examine MDMA-assisted therapy in those with schizophrenia spectrum disorders. In designing future trials, it will be important to carefully weigh the risks and benefits of the use of MDMA in individuals with schizophrenia. Those not on active antipsychotic treatment or with high positive symptom burden, high lifetime use of psychostimulants, cardiovascular diathesis, or neurologic disease should not be included in early feasibility studies until safety is better understood. The extent of negative symptoms should be considered, as well as any history of limited efficacy of standard treatments, as described previously. The use of antipsychotic medications for positive symptoms, with their known neurologic, cardiovascular, and metabolic side effects, serves as an example of how the use of compounds like MDMA for negative symptoms can be viewed in regard to risks and benefits [162]. For example, clozapine, an SGA, requires a risk–benefit analysis and weekly monitoring for months to years in order to assess for abnormal white blood cell counts due to the risk of agranulocytosis [163]. It also carries significant risks of seizure and cardiotoxicity [164,165]. Despite these known risks, it is considered a standard pharmacologic intervention for treatment-resistant schizophrenia, targeting mostly positive symptoms [164]. Both positive and negative symptoms, when untreated, bear significant functional burden [1,2,7,44]. A critical distinction in the treatment paradigm, however, is that psychedelic compounds such as MDMA are intended to be used infrequently and in the context of psychotherapy, as noted in present clinical trials. This is due to MDMA and other psychedelic compounds’ unique, long-lasting neuropsychopharmacological effects that continue beyond the transient effects noted during acute administration [154,166,167]. This is in stark contrast to the chronic daily use of antipsychotics where direct observation by a clinician may occur at long intervals.

## 5. Conclusions

In schizophrenia, negative symptoms remain a critical issue with no effective treatments. This unmet need has serious implications for individuals with schizophrenia, the field of medicine, and society at large. MDMA-assisted therapy has been heavily researched for other psychiatric indications and is likely to receive approval for the treatment of PTSD. Given the rationale presented above, MDMA-assisted therapy represents a novel modality worth investigating for negative symptoms of schizophrenia. 

## Data Availability

Not applicable.
